# Normalising comparative effectiveness trials as clinical practice

**DOI:** 10.1186/s13063-021-05566-1

**Published:** 2021-09-15

**Authors:** Tom Briffa, Tanya Symons, Nikolajs Zeps, Nicola Straiton, William Odita Tarnow-Mordi, John Simes, Ian A. Harris, Melinda Cruz, Steven A. Webb, Edward Litton, Alistair Nichol, Christopher M. Williams

**Affiliations:** 1 University of Western Australia, Perth, Western Australia Australia; 2 University of Sydney, Sydney, New South Wales Australia; 3 Epworth HealthCare, Richmond, Victoria Australia; 4 Australian Clinical Trials Alliance, Suite 1, Level 2, 24 Albert Road, Melbourne, VIC 3205 Australia; 5 Ingham Institute, Liverpool, New South Wales Australia; 6 University of New South Wales, Sydney, New South Wales Australia; 7 Monash University, Melbourne, Victoria Australia; 8 Fiona Stanley Hospital, Murdoch, Western Australia Australia; 9 University of Newcastle, Callaghan, New South Wales Australia

**Keywords:** Comparative effectiveness, Pragmatic, Trials, High-quality evidence, Clinical care, Embed

## Abstract

There is a lack of high-quality evidence underpinning many contemporary clinical practice guidelines embedded in the healthcare systems, leading to treatment uncertainty and practice variation in most medical disciplines. Comparative effectiveness trials (CETs) represent a diverse range of research that focuses on optimising health outcomes by comparing currently approved interventions to generate high-quality evidence to inform decision makers. Yet, despite their ability to produce real-world evidence that addresses the key priorities of patients and health systems, many implementation challenges exist within the healthcare environment.

This manuscript aims to highlight common barriers to conducting CETs and describes potential solutions to normalise their conduct as part of a learning healthcare system.

## Background

Many recommendations contained in contemporary clinical practice guidelines (CPG) are not supported by high quality evidence [1]. In Australia, almost three in ten evidence statements covering 748 graded recommendations from ten CPGs are low quality or consensus-based. Less than half are based on high quality evidence derived from randomised controlled trials (RCTs) or systematic review of these trials. Further afield, the picture is even more concerning. In many medical specialities, in North America and Europe, less than 20% of recommendations are supported by high quality evidence [1–3] and in specialities such as cardiology, little has changed in the last 10 years [3]. As a result, clinicians, patients, health systems and the public are unable to determine the comparative safety and efficacy of accepted treatments [4]. Clearly, more progressive approaches to evidence generation are needed to expand and strengthen the quality of recommendations in CPGs.

**Fig. 1:**
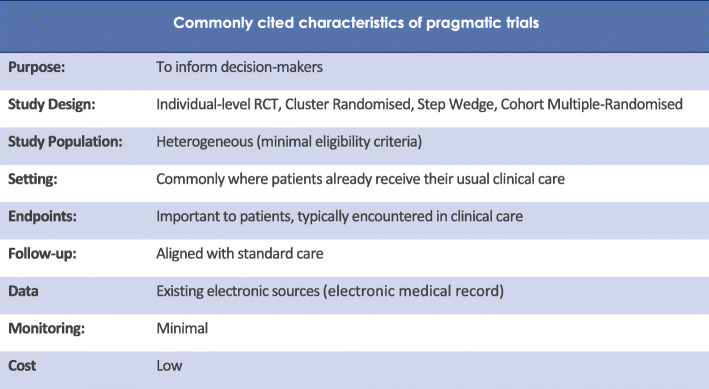
Key features of pragmatic comparative effectiveness trials

Comparative effectiveness trials (CETs) address this conundrum by randomising participants to usual care alternatives in order to generate unbiased, high-quality evidence of the relative effectiveness of existing treatments [5]. Their purpose is to assist clinicians, healthcare providers, policy makers and patients make informed decisions that improve health care at both the individual and population level. CETs often adopt pragmatic designs, and their key features are illustrated in Fig. 1.

As the treatment options investigated are widely used in practice, the additional risk to patients is low. Furthermore, public opinion polls repeatedly confirm that patients want their health systems to be ‘research active’ so that they are better informed about the benefits and risks of different treatments [4–6]. It seems likely, therefore, that working to normalise the conduct of CETs in clinical practice will help strengthen the clinician-patient relationship, especially when health systems are encouraged to partner with consumers to create learning healthcare systems (LHS), where the conduct of CETs and other high-quality research is routine, embedded and continuous [7]. The concept of an LHS was first introduced by the Institute of Medicine [8] to combat the unsustainable growth in US healthcare spending, but despite CETs being considered an essential component of an LHS, their embedding into routine practice has proven challenging. This perspective illustrates these barriers and summarises commentary on the mechanisms that could support their embedding into the health system.

Many factors have stymied attempts to integrate CETs into clinical care. Indeed, the characteristics that support their pragmatism can also hamper their conduct. For example, to preserve external validity, CETs necessarily avoid certain design features required for high internal validity, (blinding, strict inclusion criteria and a highly controlled setting) [9]. However, the large sample sizes required to account for this make some trials prohibitively expensive. In response trialists have dispensed with the costly, parallel infrastructure that is set up for each trial and instead, collect data straight from medical records and utilise front-line clinicians to undertake study activities such as informed consent. But front-line clinicians can be reticent to involve themselves in trials, partly because there are few financial (or other) incentives, and partly because they fear that such involvement may overly disrupt their clinical workflows. Very few, therefore, recommend trial participation to their patients leading to a lack of trial awareness amongst the population.

Health system leaders too are reluctant to allocate resources to support trial activity, perhaps because the research agenda has historically been driven by academics and the trials conducted within their institutions have not always been in line with health system priorities.

One of the most significant barriers to the adoption of CETs for contemporary evidence generation is the absence of proportionate approaches to trial governance.

Currently, the policy framework and governance arrangements applied to CETs are largely identical to those for trials involving novel interventions. For example, some CETs compare interventions in which verbal consent to treatment would have been obtained outside the trial setting. Yet, when formally comparing two standard treatments, lengthy, legalistic consent forms are mandated, despite growing evidence that patients prefer simpler ways to be informed about such research that do not overinflate the perception of risk [10, 11]. Therefore, a range of consent models that reflect the extent to which a trial departs from usual care seem appropriate. Some countries, including Australia, endorse a proportionate approach to trial consent [9] but uptake is still low due to fear of censure.

## Solutions to normalise the conduct of CETs

Efforts to develop, design and conduct CETs will continue to require creative approaches to overcome common challenges. Table 1 describes commentary on these challenges and possible solutions so enable the embedding of CETs into the health system.

**Table 1:** Solutions for embedding CETs

Barriers to embedding	Possible solutions
The lack of a culture of continuous improvement where knowledge creation is explicitly recognised as a core activity of the health system	- Introduce health system accreditation acknowledging centres of excellence based on indicators of research activities. - Incentivise Chief Executive Officers and board members to advance the role of research in their organisations. - Improve health system leader buy-in by more closely aligning research activity with health system priorities. - Improve stakeholder buy-in by structuring learning to focus on the outcomes of care delivery, increasing return on investment, and decreasing costs. - Engage all stakeholders to create widespread awareness of the pivotal role clinical research plays in the generation of high-quality evidence and improved health outcomes. - Restructure workflow to incorporate research activity with protected time and provide incentives for clinicians who contribute to research-driven clinical improvements. - Support studies conducted by investigator networks to address real-world evidence gaps.
Failure to apply risk-proportionate governance practices to CET	- Ensure proportionate approaches to trial ethics and governance approval that expedite study approval when trial risks are low - Utilise existing flexibilities in trial regulation to improve trial conduct (e.g. the use of flexible, patient-supported approaches to informed consent). - Ensure governance arrangements for CETs balance a trial’s risk against the risks posed by current, unresearched care. - Ensure that patients are involved in trial governance decisions and the design of research ethics and regulatory systems.
Difficulties designing and conducting large, efficient CETs	- Harmonise requirements for trial conduct to facilitate international collaboration. - Ensure trial networks collaborate with consumers and frontline clinicians to share expertise and knowledge and to drive more efficient trial practices. - Utilise novel trial designs that minimise disruption to clinical workflows. - Utilise trial platforms to create a coordinated approach to evidence generation - Incorporate implementation science strategies into CETs to facilitate uptake of evidence-based practice. - A cost-effectiveness analysis into, or alongside trials.
Limited engagement from patients and the public as active partners in advancing the delivery of care	- Introduce health system accreditation for ‘partnering with consumers’ to develop clinical trials operations so that research policies and practices reflect the needs and preferences reflect the needs and preferences of service users. - Co-design with patients a communication strategy to widely broadcast research as a core activity of the health system and that data and tissue is used to improve care within a framework that safeguards privacy and confidentiality. - Assist/enable patients to prioritise research questions so that the questions investigated, and the outcomes chosen are those most important to them. - Involve patients in the design of research so that it is conducted in ways that are sensitive to participants’ needs (e.g. by minimising the burden of participation).

Adapted from Symons et al. 2021. Making the move to a learning healthcare system: has the pandemic brought us one step closer? (10.1071/AH21076)

## Conclusion

The failure of health systems to ground everyday treatments in sound science impacts the quality of care provided to patients and contributes to health system waste. Conducting CETs that provide robust and generalisable evidence to guide practice is a public good. Importantly, patients recognise this and consistently confirm that they want their health systems to be research-active.

Although some progress has been made, few healthcare organisations have successfully transitioned into LHSs that conduct trials as an integral part of the delivery of high-quality care. Despite progress in areas such as trial design, lingering challenges remain, not least, the need to develop risk-proportionate regulatory and governance frameworks that facilitate the conduct of large, pragmatic CET by them being less expensive and cumbersome to conduct.

Implementing solutions that address the factors that currently prevent the embedding of CETs as a core activity of the health system will require disruptive change, but without such change, the widespread use of low-value or suboptimal treatments will remain unchecked.

## Data Availability

Not applicable.
